# The Impact of Ambient Temperature on Electrothermal Characteristics in Stacked Nanosheet Transistors with Multiple Lateral Stacks

**DOI:** 10.3390/nano13222971

**Published:** 2023-11-18

**Authors:** Peng Zhao, Lei Cao, Guilei Wang, Zhenhua Wu, Huaxiang Yin

**Affiliations:** 1 Integrated Circuit Advanced Process R&D Center, Institute of Microelectronics of the Chinese Academy of Sciences, Beijing 100029, China; zhaopeng@ime.ac.cn (P.Z.); caolei@ime.ac.cn (L.C.); wuzhenhua@ime.ac.cn (Z.W.); 2 State Key Lab of Fabrication Technologies for Integrated Circuits, Institute of Microelectronics of the Chinese Academy of Sciences, Beijing 100029, China; 3 School of Integrated Circuits, University of Chinese Academy of Sciences, Beijing 100049, China; 4 Process Integration, Beijing Superstring Academy of Memory Technology, Beijing 100176, China; guilei.wang@bjsamt.org.cn

**Keywords:** nanoscale device, nanosheet, self-heating effect (SHE), ambient temperature, multiple lateral stacks, thermal crosstalk

## Abstract

With characteristic size scaling down to the nanoscale range, the confined geometry exacerbates the self-heating effect (SHE) in nanoscale devices. In this paper, the impact of ambient temperature (*T*_amb_) on the SHE in stacked nanosheet transistors is investigated. As the number of lateral stacks (*N*_stack_) increases, the nanoscale devices show more severe thermal crosstalk issues, and the current performance between n- and p-type nanoscale transistors exhibits different degradation trends. To compare the effect of different *T*_amb_ ranges, the temperature coefficients of current per stack and threshold voltage are analyzed. As the *N*_stack_ increases from 4 to 32, it is verified that the zero-temperature coefficient bias point (*V*_ZTC_) decreases significantly in p-type nanoscale devices when *T*_amb_ is above room temperature. This can be explained by the enhanced thermal crosstalk. Then, the gate length-dependent electrothermal characteristics with different *N*_stack_s are investigated at various *T*_amb_s. To explore the origin of drain current variation, the temperature-dependent backscattering model is utilized to explain the variation. At last, the simulation results verify the impact of *T*_amb_ on the SHE. The study provides an effective design guide for stacked nanosheet transistors when considering multiple stacks in circuit applications.

## 1. Introduction

With the characteristic size of integrated circuits (ICs) scaling down to the nanoscale range, the core transistor structures have gradually evolved into gate-all-around nanosheet FET (GAA NSFET) [1]. The GAA structure exhibits excellent electrostatic performance compared to FinFET technology [2]. At the same time, the nanosheet channel with a vertically stacked structure shows great performance advantages [3]. However, the confined geometry and low thermal conductivity materials, such as gate oxide and HfO_2_, greatly hinder heat transport, leading to a severe self-heating effect (SHE) [4,5,6,7]. The thermal conductivity of Si active regions decreases significantly due to intensified phonon scattering. Then, the thermal conductivity of the source/drain (S/D) causes an additional decrease due to heavy doping and the SiGe alloy material adopted. In addition, the nanosheet channels are floated and isolated from the substrate, making it difficult to transport heat. Due to the gate stack structure, phonon-boundary scattering is intensified. These factors lead to SHE issues being prominent in NSFET. The SHE can cause degradation of electrical performance and bring about reliability issues, ultimately decreasing the device’s lifetime significantly [8,9,10,11,12]. Some researchers have conducted a series of investigations into SHE issues, including compact models, optimization technologies, and self-heating mechanisms [13,14,15,16,17,18,19]. In addition, the multiple lateral stacks are fabricated in NSFETs for high performance. The self-heating will be exacerbated due to the rising current in the lateral stack structure [7]. This is one aspect of thermal issues.

On the other hand, the core transistors work in practical application circuits. The electrical performance is inevitably affected by ambient temperature (*T*_amb_), where the *T*_amb_ includes those from inside the chip and the surrounding environment. The high *T*_amb_ can change the temperature-sensitivity threshold voltage (*V*_th_) and introduce variability in the on-state current. At the same time, the *T*_amb_ plays a crucial role in thermal properties, such as thermal conductivity, lattice temperature rise, and thermal resistance (*R*_th_) [20,21,22,23,24,25]. Some researchers have analyzed the electrothermal characteristics of FinFETs with multiple fins under the impact of *T*_amb_ [20,23,26]. However, few studies focus on the research of *T*_amb_ on NSFET with different numbers of lateral stacks (*N*_stack_). In addition, there are few works on the geometry effect in NSFETs with different *N*_stack_s. Therefore, we performed a symmetrical investigation on the electrothermal performance of NSFETs with different *N*_stack_s under the *T*_amb_ impact. Furthermore, the geometry effect with different *N*_stack_s is also studied.

In this paper, the *T*_amb_-dependent SHE in NSFETs with different *N*_stack_s is symmetrically measured and analyzed. The rest of the article is divided into three parts. Section 2 discusses the fabrication of NMOS and PMOS in detail. In Section 3, the current variation with different *N*_stack_s is explored. Then, the geometry effect with different gate lengths is further analyzed based on the backscattering model. In addition, the simulations are conducted for verification. Finally, the conclusion is given in Section 4.

## 2. Device Fabrication

**Figure 1:**
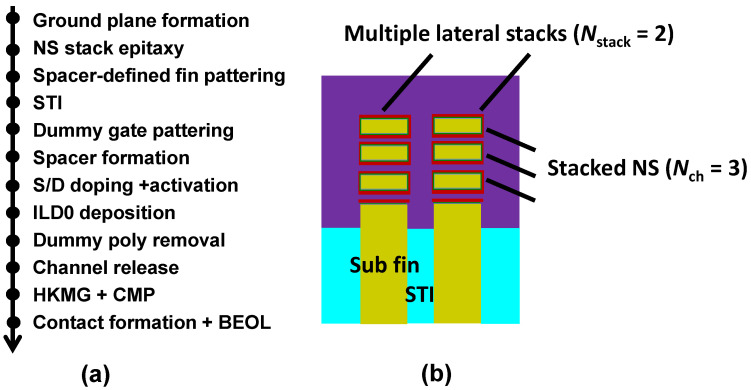
(**a**) Process flow of GAA NSFETs adopting the gate last process. (**b**) Schematic of the NSFET with multiple lateral stacks along the nanosheet width direction.

The integration process flow design of the NSFET is summarized in Figure 1a, where the NSFET was grown on {100} bulk Si substrates. This process flow is based on the conventional fabrication process [27]. The fabrication adopts the gate last process. Before the stacked nanosheets were formed, B and P were implanted to suppress the bottom parasitic channel in the ground plane process for NMOS and PMOS, respectively [28]. The stacked GeSi/Si layers were formed using the epitaxy process. The nanosheet channels are stacked vertically with three layers (*N*_ch_ = 3). In this step, the reduced pressure chemical vapor deposition was used to grow the periodical GeSi/Si multilayer with 16 nm Ge_0.3_Si_0.7_ and 10 nm Si. Then, the fin array was carried out using spacer image transfer (SIT) technology, which was formed using a SiN_x_ hard mask. For high-performance desire, the stacked nanosheets were fabricated with multiple lateral stacks in this step, as shown in Figure 1b. The number of parallel nanorails of lateral stacks is defined as *N*_stack_. The *N*_stack_ is 2, 4, 8, 16, and 32. Next, the shallow trench isolation (STI) was formed to decrease the leakage current between the devices. Meanwhile, the rapid annealing process was performed to make the film compact. Then, a dummy gate was fabricated with amorphous Si to protect the nanosheets. After the deposition of amorphous Si, the chemical mechanical planarization (CMP) was used to perform the planarization process. The dummy gate image was formed using deposition and etch of Si_3_N_4_ hard mask. The inner spacer formation was a key process that was deposited using SiN_x_ and etched using reactive ion etching (RIE). After this, the in-situ doping process with highly doped doses and the activation process were carried out to form S/D. The zero-level interlayer dielectric (ILD0) with SiN_x_ is used to avoid the over-etch phenomenon. After the dummy gate removal, GeSi was selectively removed using the wet-etched technique. After forming the thin gate oxide layer, the HfO_2_ was deposited and the nanosheet was surrounded by different metal stacks. The atom-layer-deposition technology (ALD) was used to deposit the multilayer HK/MG films, and the CMP was performed to achieve gate separation. Finally, the tungsten contact and the BEOL process were fabricated to complete the NSFETs.

**Figure 2:**
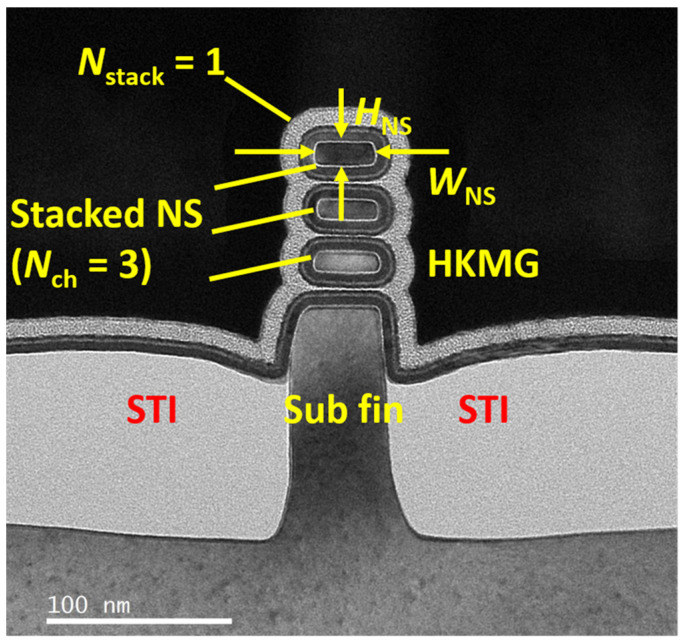
The TEM image of GAA NSFETs in this work. The nanosheets are separated from each other.

**Figure 3:**
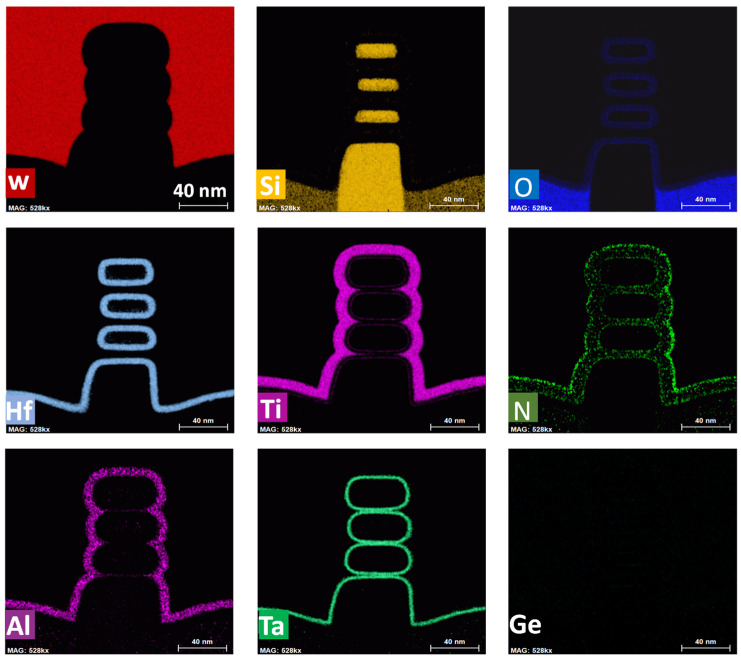
The EDS images of NSFETs in this work. The top figures illustrate W, Si, and O elements. The middle figures illustrate Hf, Ti, and N elements. The bottom figures illustrate Al, Ta, and Ge elements. Ge has been removed completely.

Through the measurement of transmission electron microscopy (TEM), Figure 2 shows the fabricated channel structure of NSFETs, which can be seen that the NSFETs are successfully fabricated according to the process flow. The nanosheets are separated using HK/MG, and the STI is formed near the sub-fin. The width of the sub-fin is similar to that of the nanosheet. The nanosheet width (*W*_NS_) is about 30 nm, and the nanosheet thickness (*T*_NS_) is about 10 nm. The gate length (*L*_G_) is 30, 40, 60, and 500 nm. Then, energy-dispersive spectroscopy (EDS) is used to exhibit the distribution of elements in NSFETs, as shown in Figure 3. It shows that Ge has been completely removed, and the nanosheets are separated from each other. Each nanosheet is surrounded by Hf and oxide elements. This indicates that the nanosheets can be well controlled using bias voltages. At the same time, it can be seen that Al, Ti, N, and Ta elements are deposited surrounding the nanosheets, and the W element has been wrapped to the Si nanosheets.

## 3. Results and Discussion

### 3.1. Electrothermal Performance of Different Numbers of Stacks under the Impact of Ambient Temperature

Since the measurement is dependent on ambient temperature (*T*_amb_), the *T*_amb_-dependent SHE in NSFETs is measured using the Agilent B1500 semiconductor parameter analyzer (Agilent Technologies, Santa Clara, CA, USA). The *T*_amb_ varies from −50 °C to 125 °C in a 25 °C step. 

**Figure 4:**
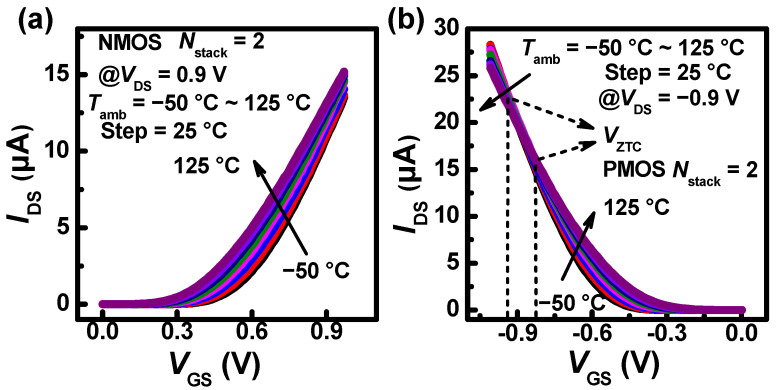
Experimental temperature dependence of transfer characteristics at *L*_G_ = 500 nm, *N*_stack_ = 2, (**a**) *V*_DS_ = 0.9 V for NMOS and (**b**) *V*_DS_ = −0.9 V for PMOS. It shows two *V*_ZTC_ under *T*_amb_ = −50~25 °C range and *T*_amb_ = 25~125 °C range.

As shown in Figure 4, the transfer characteristic curves exhibit a variation in drain current (*I*_DS_) as the *T*_amb_ increases for NMOS and PMOS. The threshold voltages (*V*_th_s) are 300 mV and −300 mV at *T*_amb_ = 25 °C of room temperature (*T*_rt_) for NMOS and PMOS, respectively. The *V*_th_ is extracted using the constant current method. As the *T*_amb_ increases, the |*V*_th_| decreases because the carrier concentration is positively correlated with the *T*_amb_. In Figure 4a, the *I*_DS_ of NMOS shows an increasing trend. Meanwhile, the *I*_DS_ of PMOS also shows an increasing trend at a lower |*V*_GS_|. However, it exhibits a reverse temperature dependence at a larger |*V*_GS_|, as shown in Figure 4b. This is because the effect of the *V*_th_ reduction exceeds that of the mobility degradation in NMOS. However, the effect of the |*V*_th_| reduction is inferior to that of the mobility degradation at a larger |*V*_GS_| in PMOS. Moreover, the *I*_DS_ of PMOS is higher than that of NMOS at a larger |*V*_GS_|. The current degradation indicates that the higher current is more severely impacted by *T*_amb_. In addition, it is found that the zero-temperature coefficient bias point (*V*_ZTC_) occurs in PMOS, where the *V*_ZTC_ represents the bias voltage point that the *I*_DS_ is independent of the *T*_amb_ [29]. In addition, there are two *V*_ZTC_ under *T*_amb_ = −50~25 °C range and *T*_amb_ = 25~125 °C range.

**Figure 5:**
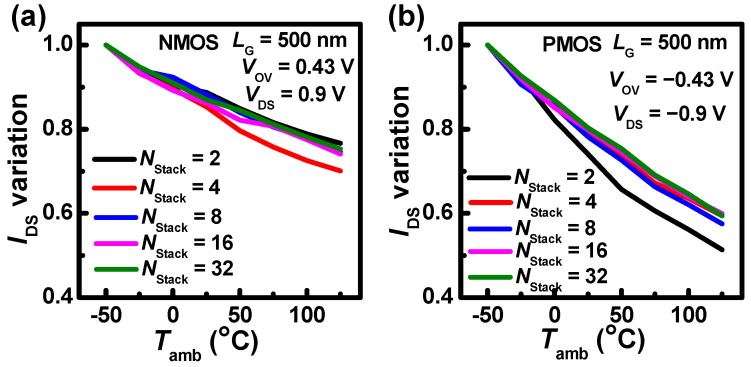
(**a**) *I*_DS_ variations of NMOS with different *N*_stack_s under the impact of *T*_amb_ relative to the *I*_DS_ at *T*_amb_ = −50 °C, (**b**) *I*_DS_ variations of PMOS with different *N*_stack_s under the impact of *T*_amb_ relative to the *I*_DS_ at *T*_amb_ = −50 °C.

To explore the *T*_amb_-dependent relation, Figure 5 shows that the *I*_DS_ variations with different *N*_stack_s under the impact of *T*_amb_ are extracted relative to the *I*_DS_ at *T*_amb_ = −50 °C, and the gate overdrive voltage (*V*_ov_ = *V*_GS_ − *V*_th_) is 0.43 V and −0.43 V for NMOS and PMOS, respectively. The *I*_DS_ degradation of NMOS is lower than that of PMOS. At high *T*_amb_, The NMOS with *N*_stack_ = 4 shows the largest *I*_DS_ degradation, and other multiple stacks have minor differences. The devices with *N*_stack_ = 2 have the lowest *I*_DS_ degradation at high *T*_amb_ in Figure 5a. PMOS shows a different trend when *N*_stack_ increases, as shown in Figure 5b. The PMOS with *N*_stack_ = 2 shows the largest *I*_DS_ degradation, and other multiple stacks have minor differences. The PMOS with *N*_stack_ = 32 shows the lowest *I*_DS_ degradation. The *I*_DS_ variation in NMOS with *N*_stack_ = 4 and PMOS with *N*_stack_ = 2 may be induced by random structure irregularities.

**Figure 6:**
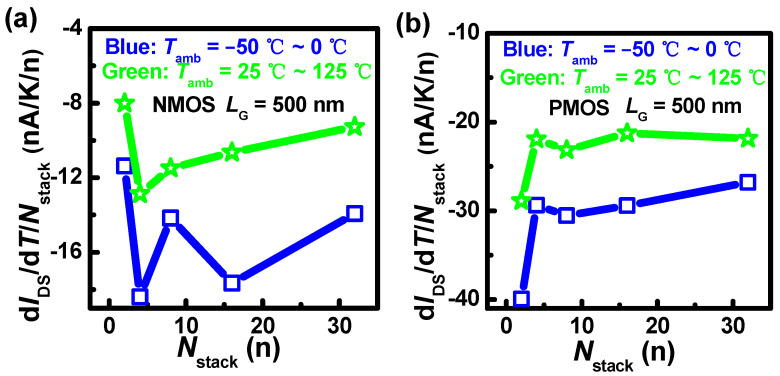
*L*_G_ = 500 nm, (**a**) *V*_DS_ = 0.9 V, *V*_ov_ = 0.59 V, the temperature coefficients of *I*_DS_ per stack in NMOS, (**b**) *V*_DS_ = −0.9 V, *V*_ov_ = −0.59 V, the temperature coefficients of *I*_DS_ per stack in PMOS under different *T*_amb_ ranges.

To analyze the origin of the *I*_DS_ variation, the temperature coefficients of *I*_DS_ per stack (β) are extracted, as shown in Figure 6. Figure 6a shows that β in NMOS decreases in the negative direction under *T*_amb_ above *T*_rt_ when *N*_stack_ is from 4 to 32. This is induced by the coupling mechanism with the impact of the SHE and *T*_amb_. As the *N*_stack_ increases, the thermal crosstalk is enhanced in stacked nanosheets, where the thermal crosstalk is part of the SHE. The SHE can intensify phonon-electron scattering, and then counteract the partial effect of *T*_amb_, resulting in reduced *I*_DS_ degradation. In Section 3.3, the simulation results will further verify the speculation. The β in NMOS with *N*_stack_ = 2 is the lowest [Figure 6a]. This can be caused by difficult heat transport due to the smaller contact area between nanosheets and the S/D region, leading to severe thermal crosstalk. Meanwhile, the β under *T*_amb_ below 0 °C is basically larger than that under *T*_amb_ above *T*_rt_. This is because the impact of thermal crosstalk is weakened by enhanced heat transport ability.

Figure 6b shows that the β in PMOS is larger than that in NMOS. The carrier scattering intensifies by *T*_amb_ because the current density (*J*_DS_) in PMOS is larger than that in NMOS. The PMOS with *N*_stack_ = 2 exhibits the largest β. This reverse behavior is due to the highest *J*_DS_ in PMOS with *N*_stack_ = 2 compared to NMOS. In addition, the difference of β when *N*_stack_ = 4~32 is small. This is because the difference in *J*_DS_ of PMOS is small, and thus the difference in thermal crosstalk is relatively insignificant.

**Figure 7:**
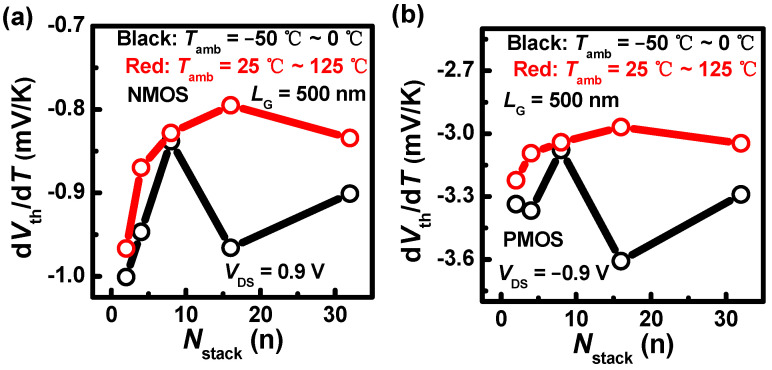
*L*_G_ = 500 nm, the temperature coefficients of *V*_th_ (η) with different *N*_stack_s in (**a**) NMOS and (**b**) PMOS under different *T*_amb_ ranges.

Then, the temperature coefficient of *V*_th_ (η) is extracted, as shown in Figure 7. The η in NMOS under *T*_amb_ above *T*_rt_ rapidly decreases in the negative direction with *N*_stack_ first, and then the velocity of decrease gradually slows down [Figure 7a]. The η of NMOS with *N*_stack_ = 16 is the lowest. This is because the thermal crosstalk becomes severe; therefore, the impact of *T*_amb_ is mitigated. However, it increases when the *N*_stack_ grows to 32. This can be explained by the heat transport ability that the contact area between nanosheets and S/D regions gradually increases with *N*_stack_, and then the enhanced thermal crosstalk effect is weakened. Therefore, the η with *N*_stack_ = 32 increases. The η in NMOS under *T*_amb_ below 0 °C rapidly decreases first, and then increases with *N*_stack_ = 16. The η in PMOS exhibits a similar trend compared with that in NMOS [Figure 7b]. However, the η value is larger.

The relation between the *I*_DS_ variation and the η based on the temperature-dependent backscattering model is also further analyzed. In the temperature-dependent backscattering model [30,31], the *I*_DS_ formula is defined using (1), and the linear relation of *I*_DS_ and *T*_amb_ is given using (2). Finally, the analytic expression for α concerning temperature is given using (3):

(1) $$I_{\mathrm{DS}}=WQ_{\mathrm{inj}}v_{\mathrm{th}}(\frac{\lambda_{0}/l_{0}}{2+\lambda_{0}/l_{0}})$$

(2) $$\Delta I_{\mathrm{DS}}/I_{\mathrm{DS}}=\alpha (T-(-50\;℃))$$

(3) $$\alpha =(\frac{1}{2}-{\frac{4}{2+\lambda_{0}/l}}_{0})/298\;K-\frac{\eta }{V_{\mathrm{GS}}-V_{\mathrm{th}0}}$$

In the equations, *Q*_inj_ is the inverse layer density near the source region; *ν*_th_ is the thermal injection velocity at the thermal source; *λ*_0_ is the mean-free path; *l*_0_ is the critical distance when the carriers travel over a *KT* layer from the thermal source, where *K* and *T* are Boltzmann constant and temperature, respectively; and *V*_th0_ is the threshold voltage at referenced temperature −50 °C. The α includes the backscattering coefficient term and the voltage-dependence term. In Figure 7, the η in PMOS is higher than that in NMOS significantly, and thus the second term of (3) becomes larger. This leads to a greater degradation of the *I*_DS_ in PMOS compared to NMOS when *T*_amb_ increases, as shown in Figure 5.

**Figure 8:**
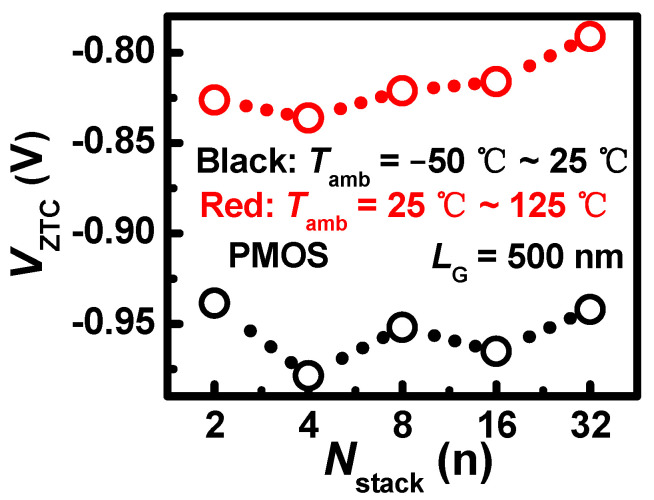
*L*_G_ = 500 nm, the *V*_ZTC_ of PMOS with different *N*_stack_ under *T*_amb_ = −50–25 °C range and *T*_amb_ = 25–125 °C range.

As a special thermal phenomenon, the *V*_ZTC_ of PMOS is extracted in Figure 8. It is shown that the *V*_ZTC_ of PMOS decreases in the negative direction under *T*_amb_ above *T*_rt_ when *N*_stack_ grows from 4 to 32. The result can be explained by the coupling mechanism with the impact of the SHE and *T*_amb_. As the *N*_stack_ increases, the current variation comes to balance at lower |*V*_GS_| due to the effect of *V*_th_ and mobility. However, the *V*_ZTC_ under *T*_amb_ below *T*_rt_ is 0.1 V higher than that under higher *T*_amb_ in the negative direction. This is because the SHE plays a lesser role in the case of *T*_amb_ below *T*_rt_. Therefore, the effect of *V*_th_ and mobility compensate at a higher *V*_GS_ for the lower *T*_amb_. The investigation of *V*_ZTC_ is useful to explore the working voltage in real applications.

### 3.2. Electrothermal Performance of Different Gate Lengths with Different N_stack_s under the Impact of T_amb_

**Figure 9:**
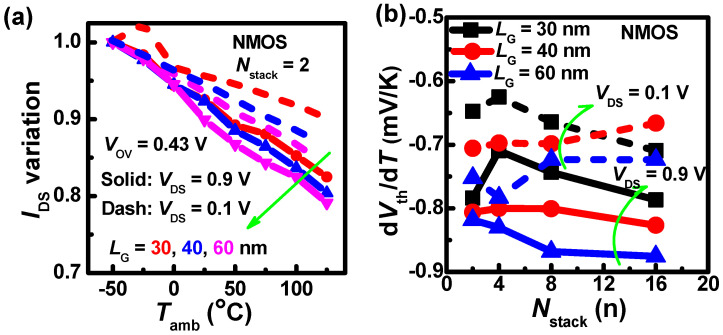
(**a**) *I*_DS_ variations with different gate lengths under the impact of *T*_amb_ relative to the *I*_DS_ at *T*_amb_ = −50 °C. (**b**) The temperature coefficients of *V*_th_ with different gate lengths when the *N*_stack_ increases.

To explore the geometry effect, we further analyze the impact of gate lengths on electrical performance with different *N*_stack_s, as shown in Figure 9. As *L*_G_ increases, the degradation of *I*_DS_ becomes severe. This can be explained by the temperature-dependent backscattering model. 

As the *L*_G_ increases, the channel potential decreases, and then *l*_0_ increases. Thus, the first term of (3) declines. This is an inverse result with the *I*_DS_ degradation [Figure 9a]. Furthermore, the η is extracted, as shown in Figure 9b. The η increases in the negative direction as the *L*_G_ increases at *V*_DS_ = 0.9 V. Therefore, the *I*_DS_ degradation is the result of both terms. At the same time, when the *N*_stack_ increases at *V*_DS_ = 0.9 V, the devices with *L*_G_ = 30, 40 nm show that the η decreases in the negative direction first, then, increases, similar to the trend of devices with *L*_G_ = 500 nm. Remarkably, when *V*_DS_ = 0.9 V, the slope of η with *N*_stack_ located in the 8 and 16 range is negative first, and then positive when the *L*_G_ increases from 30 nm to 500 nm.

In addition, the *I*_DS_ degradation in short gate length devices at *V*_DS_ = 0.9 V is higher than that at *V*_DS_ = 0.1 V. The difference gradually becomes larger with *T*_amb_, and the device with *L*_G_ = 30 nm has the largest difference. Figure 9b shows that the η increases in the negative direction as the *V*_DS_ increases, and thus the second term of (3) becomes larger. Then, the *λ*_0_/*l*_0_ also increases with larger *V*_DS_, and the first term of (3) becomes larger. Finally, the *I*_DS_ degradation is higher with larger *V*_DS_.

### 3.3. Simulation Verification

**Figure 10:**
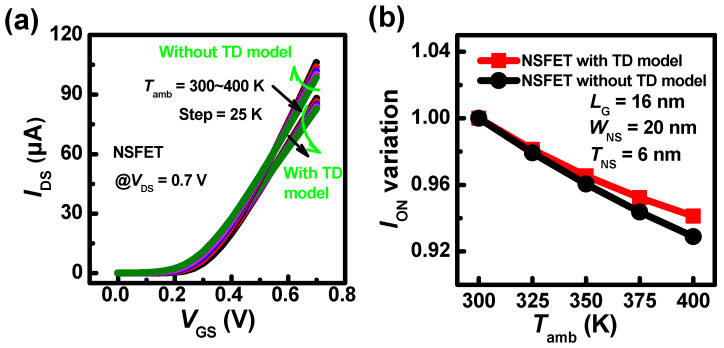
(**a**) The transfer characteristics of NSFET (*N*_stack_ = 1) with/without the SHE as the *T*_amb_ increases from 300 K to 400 K, (**b**) *I*_DS_ variations relative to the *I*_DS_ at *T*_amb_ = 300 K with/without the SHE when *V*_GS_ = *V*_DS_ = 0.7 V.

**Figure 11:**
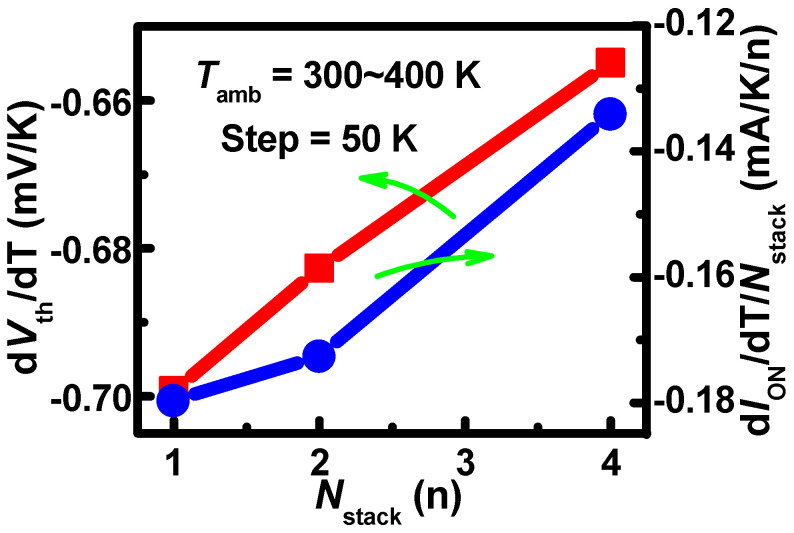
The temperature coefficients of *V*_th_ and the temperature coefficients of *I*_DS_ per stack with different *N*_stack_s.

The coupling mechanism with the impact of the SHE and *T*_amb_ is verified using the simulation method. To clarify the SHE clearly, the 16 nm gate length devices are simulated. The simulated devices are 3 nm node NSFETs, referring to [1]. The electrothermal parameters are set according to [32], where the *L*_G_, *W*_NS_, and *T*_NS_ are set to 16, 20, and 6 nm, respectively. The nanosheets adopt three layers of vertically stacked structure. All simulations are performed using Sentaurus TCAD tools [33]. The SHE is calculated with the thermodynamic model (TD model). Figure 10a shows that the *I*_DS_ with the SHE is lower than that without the SHE at larger *V*_GS_. At *V*_GS_ = *V*_DS_ = 0.7 V, the on-state *I*_DS_ (*I*_ON_) degradation with the SHE is lower than that without the SHE as *T*_amb_ increases, as shown in Figure 10b. The results indicate that the SHE weakened the impact of *T*_amb_. As the *N*_stack_ increases, the η declines in the negative direction as shown in Figure 11. At the same time, the β decreases with *N*_stack_. This further verifies that the coupling heat counteracts the partial effect of *T*_amb_.

To further investigate thermal characteristics, the thermal resistance (*R*_th_) and the maximum lattice temperature rise (∆*T*_max_) with respect to *T*_amb_ are extracted. The *R*_th_ is defined using (4), where total heat includes Joule heat, Peltier heat, Tompson heat, and recombination heat [34].

*R*_th_ = ∆*T*_max_/total heat (K/μW) (4)

**Figure 12:**
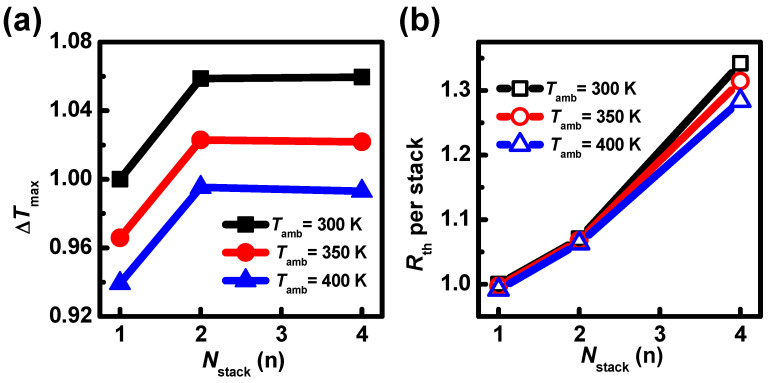
When *V*_GS_ = *V*_DS_ = 0.7 V, (**a**) ∆*T*_max_ and (**b**) *R*_th_ variations relative to the devices with *N*_stack_ = 1 under *T*_amb_ = 300 K.

The ∆*T*_max_ and *R*_th_ in the devices with *N*_stack_ = 1 under *T*_amb_ = 300 K are 149 K and 2.59 K/μW, respectively, as shown in Figure 12. In Figure 12a, the ∆*T*_max_ decreases with *T*_amb_. However, the lattice temperature gradually increases. This explains why *I*_DS_ with the SHE is lower at larger *V*_GS_ [Figure 10a]. At the same time, the ∆*T*_max_ increases when *N*_stack_ is from 1 to 2 first, and then has a minor variation when *N*_stack_ is from 2 to 4. This can be explained by the *R*_th_ variation with *N*_stack_. In Figure 12b, the *R*_th_ per stack decreases with *T*_amb_. This is because the ∆*T*_max_ reduction ratio exceeds the *I*_ON_ degradation. Then, the *R*_th_ per stack increases when *N*_stack_ is from 1 to 2, and then the rising speed increases when *N*_stack_ is from 2 to 4. This is induced by the coupling effect. The rising *R*_th_ causes an increase in lattice temperature, leading to a decrease in current density, eventually, the ∆*T*_max_ variation is low when *N*_stack_ is from 2 to 4 compared to that when *N*_stack_ is from 1 to 2. In addition, when the *T*_amb_ increases, the *R*_th_ decreases significantly in the devices with *N*_stack_ = 4 compared to that with *N*_stack_ = 1 and 2. This is because the coupling heat is more severe in the devices with *N*_stack_ = 4. These results verify that the SHE counteracts the partial effect of *T*_amb_.

## 4. Conclusions

In this paper, the impact of *T*_amb_ on the SHE in NSFET with different *N*_stack_s is investigated. The results show that the NMOS with *N*_stack_ = 2 has the lowest *I*_DS_ degradation, and the *I*_DS_ degradation of PMOS with *N*_stack_ = 32 is lower than that with *N*_stack_ = 2. The results show that the *I*_DS_ degradation is lowest in the NMOS with *N*_stack_ = 2 and the PMOS with *N*_stack_ = 32 when the *T*_amb_ is at a high level. Due to the coupling mechanism with the impact of the SHE and *T*_amb_, the η exhibits a decrease trend first, and then an increase trend with *N*_stack_ when the *T*_amb_ is higher than *T*_rt_. Remarkably, the *V*_ZTC_ of PMOS decreases with *N*_stack_ > 4 in the negative direction when *T*_amb_ is higher than *T*_rt_. Based on the backscattering theory, the *I*_DS_ degradation ratio decreases when the *L*_G_ becomes shorter. Meanwhile, the *I*_DS_ degradation decreases at *V*_DS_ = 0.1 V compared to that at *V*_DS_ = 0.9 V in short gate length devices. Finally, the simulations verify that the SHE counteracts the partial effect of *T*_amb_. The work explores the electrothermal characteristics when NSFETs with different *N*_stack_s work under the impact of *T*_amb_ and provides design guidelines for real applications.

## Data Availability

The data presented in this study are available on request from the corresponding author.
